# Massive Pulmonary Embolus Following a Hemorrhagic Stroke: A Thrombolysis Therapy Dilemma

**DOI:** 10.7759/cureus.37816

**Published:** 2023-04-19

**Authors:** Olayemi J Akanmode, Abiodun M Akanmode, Rahman A Olusoji, Akolade S Osanoto

**Affiliations:** 1 Hyperacute Stroke Unit, Internal Medicine, King's College Hospital NHS Foundation Trust, London, GBR; 2 Internal Medicine, Columbia University College of Physicians and Surgeons, Harlem Hospital Center, New York, USA; 3 General Practice, Windsor University School of Medicine, Basseterre, KNA

**Keywords:** hemorrhagic stroke, pulmonary embolus, pulmonary embolism, neurosurgery, medical icu, pulmonary embolism (pe), systemic thrombolysis, stroke

## Abstract

Pulmonary embolism varies in presentation with factors such as embolus size and pre-existing comorbidities contributing significantly. Despite the availability of several options to treat pulmonary embolism, these options significantly decrease when a massive pulmonary embolism causes a cardiac arrest in the setting of a recent hemorrhage thalamic stroke. We reviewed the current literature and presented a case report. In addition, we presented seven cases of pulmonary embolus where thrombolysis was used despite an absolute contraindication to thrombolysis, and the patients had successful outcomes.

## Introduction

Current guidelines recommend thrombolytic therapy for cases of massive pulmonary embolus (PE) without any contraindications. However, thrombolysis is contraindicated in the presence of a recent hemorrhagic stroke or other conditions [1]. Some investigators argue that the current contraindications to thrombolysis have been extracted from data from acute coronary syndrome and can be viewed as relative rather than absolute contraindications, especially with cases of massive PE associated with high mortality [2].

We present a case of a 75-year-old man who had earlier suffered a hemorrhagic stroke (an absolute contraindication to thrombolysis) and developed a massive PE complicated by cardiac arrest with pulseless electrical activity. Despite current practices and guidelines, we administered thrombolytics successfully to the patient with no further intracranial bleeding.

Hemodynamic compromise secondary to massive PE is associated with a mortality rate of about 50-100%. In these cases, emergency thrombolysis is required, as suggested by current guidelines; however, there is no clear-cut therapeutic guideline when there is an absolute contraindication to thrombolysis. Hence, when faced with an absolute contraindication dilemma, an individual approach to thrombolysis should be taken [3].

## Case presentation

**Figure 1:**
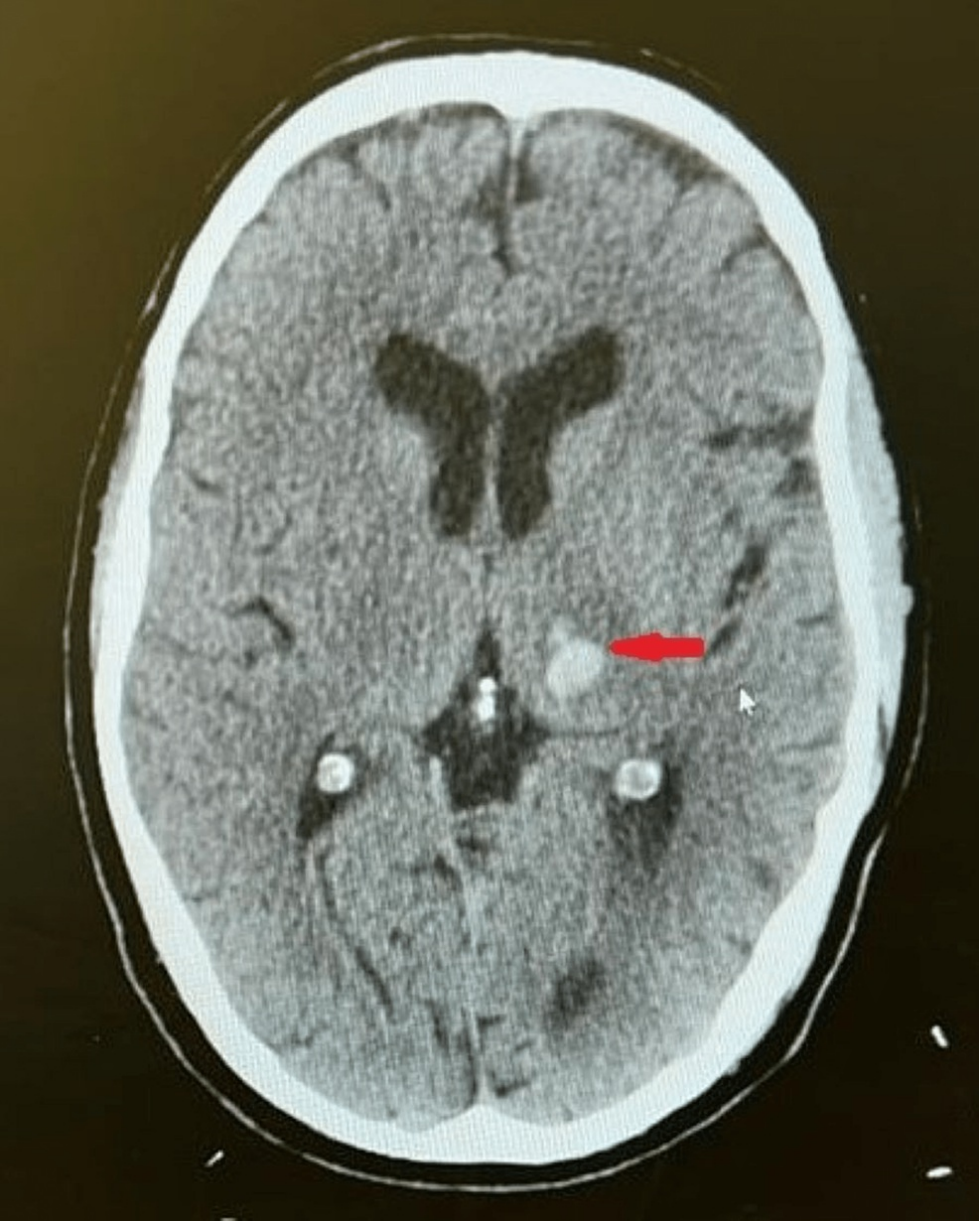
Axial image of a non-contrast CT of the brain showing hyperdense left-sided intracranial hemorrhage (red arrowhead) measuring 10 x 12 mm with surrounding small volume edema. There is no mass effect and no associated midline shift.

A 75-year-old man arrived at the emergency department with sudden onset right-sided weakness. His past medical history included essential hypertension, alcohol dependence, and asthma. Although the patient's hypertension was managed with amlodipine 10 mg daily and ramipril 5 mg daily, adherence with medications and regular checkups were not optimal, and he had no allergies. On admission, a physical examination revealed right facial weakness, slurred speech, right arm and leg weakness, and right-sided sensory deficit. Blood pressure was 214/95 mmHg, the temperature was 36.4°C, and the respiratory rate was 18 breaths per minute. The chest was clear, the abdomen was soft and non-tender, heart sounds were normal, and the calves were soft and non-tender. Initial routine laboratory tests were within the normal range, including complete blood count, renal function, liver function panel, and coagulation screen. The international normalized ratio was 1.0. An initial electrocardiogram (EKG) showed normal sinus rhythm. Initial imaging included a non-contrast CT scan that revealed a 14 mm left thalamic parenchymal hemorrhage with surrounding small-volume edema (Figure 1). The neurosurgical team was immediately involved, and they advised conservative management. We transferred our patient to the hyperacute stroke unit, where he received continuous care. Blood pressure control was optimized with home meds, and he remained hemodynamically stable. We applied intermittent compression stockings for venous thromboembolism prophylaxis.

**Figure 2:**
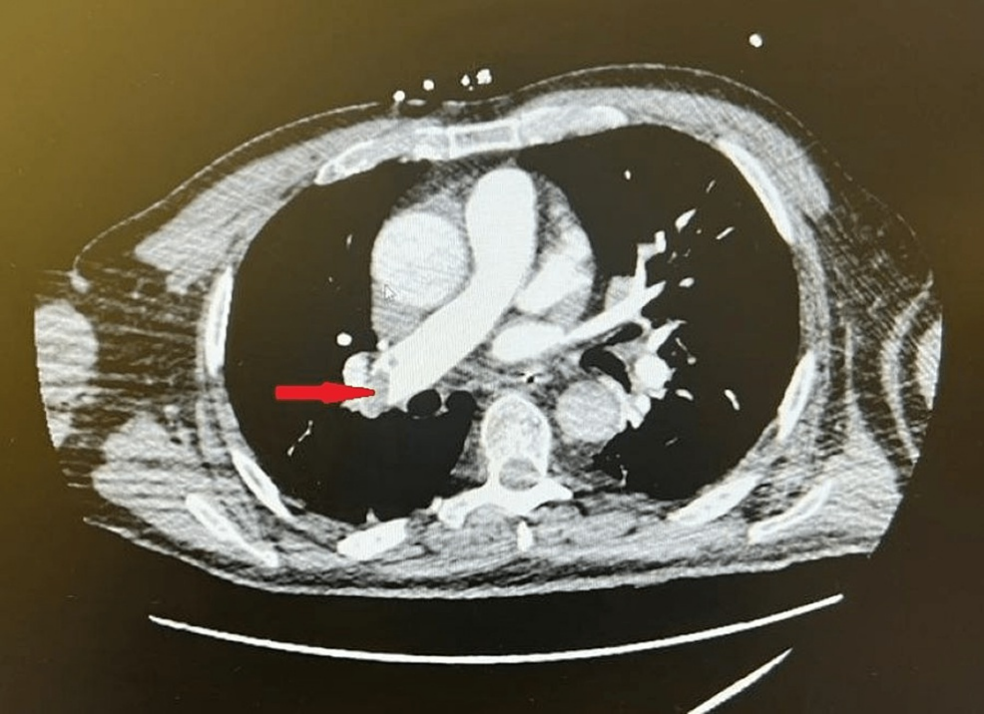
Axial image of the CT of the chest with intravenous contrast showing the right main pulmonary artery embolisms (red arrowhead).

On the second day after admission, the physiotherapist provided neuro and limb therapy to the patient. The patient's vital signs were recorded and showed tachycardia with a heart rate of 110 beats per minute, a respiratory rate of 22 breaths per minute, and a blood pressure of 90/50 mmHg. During re-evaluation, the patient suddenly became unresponsive, and the medical team initiated high-quality cardiopulmonary resuscitation (CPR) after activating the cardiac arrest team. A rhythm check was performed, which showed pulseless electrical activity. The medical team achieved a spontaneous return of circulation through CPR, and they intubated the patient, provided mechanical ventilation, and started administering norepinephrine for vasopressor support. The patient's laboratory results were normal except for the D-dimer, which showed a value of 22,859 ng/ml. A CT pulmonary angiogram revealed an extensive acute pulmonary embolism with evidence of right heart strain (Figure 2).

**Figure 3:**
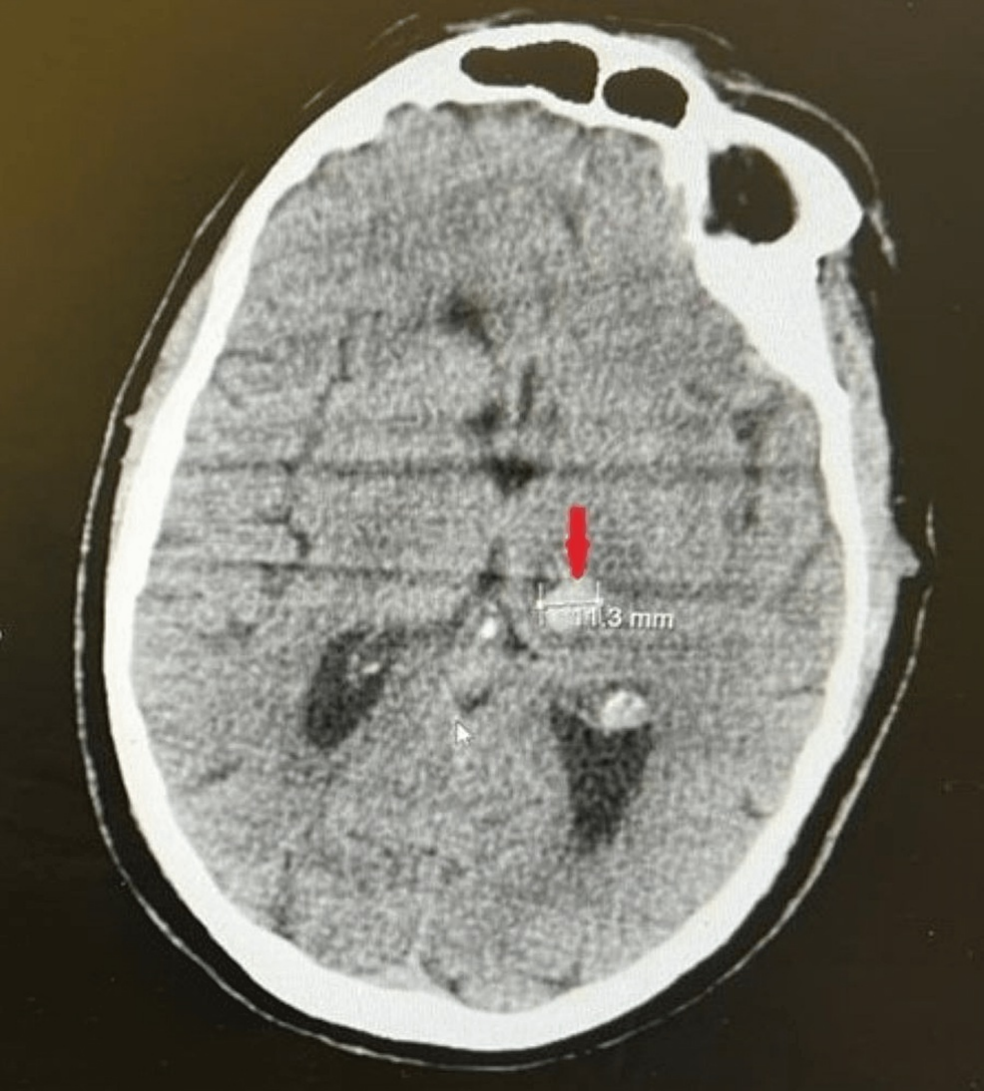
Axial image of a non-contrast CT of the brain post thrombolysis showing the same hyperdense left-sided intracranial hemorrhage (red arrowhead) measuring 11.3 mm with surrounding small volume edema indicating that there was no worsening or expansion of the intracranial bleed after alteplase administration.

A multidisciplinary meeting was held between the intensive care unit (ICU) team, stroke team, and hematology team, and they decided to administer alteplase to the patient due to the high risk of mortality. The patient was thrombolyzed with alteplase and stabilized before being transferred to the ICU. A repeat CT scan of the head was performed to evaluate the state of the left thalamic parenchymal hemorrhage, and it showed no increase in the bleed size (Figure 3).

The patient continued to receive care in the ICU and remained hemodynamically stable with no further indications for repeat neuroimaging. On day five of admission, he began to deteriorate. He was noted to be desaturating to the 80s while ventilated and having a low-grade fever of 37.7°C. A sepsis workup was performed, including blood cultures, chest X-ray, inflammatory marker (C-reactive protein), and complete blood count. Broad-spectrum antibiotic therapy with piperacillin-tazobactam was initiated for possible hospital vs. ventilator-associated pneumonia. Unfortunately, the patient passed away on day seven of hospitalization with the formal cause of death being sepsis secondary to pneumonia.

## Discussion

To manage acute massive pulmonary embolism, prompt treatment with thrombolytics is crucial. However, when a patient has recently experienced intracranial hemorrhage complicated by a massive pulmonary embolism leading to cardiac arrest, there is a dilemma on how to proceed.

Massive pulmonary embolism, which involves right ventricular failure associated with a systolic blood pressure drop of less than 90 mmHg or a pressure drop of less than 40 mmHg lasting for at least 15 minutes, is a life-threatening condition that requires immediate thrombolytic treatment. However, according to current guidelines, thrombolytics are contraindicated for patients with a history of central nervous system lesions, recent brain or spine surgery, major trauma, active bleeding, or ischemic or hemorrhagic stroke [4]. Thrombolysis is also not recommended in patients with direct infusion of thrombolytic agents. It does not improve the thrombolysis rate and is associated with bleeding complications at the catheter site [5].

Endovascular clot retrieval or surgical embolectomy is another option, but it requires skill and expertise and carries the risk of prolonged bleeding at the catheter insertion site. Patients who have undergone cardiopulmonary resuscitation have a higher mortality risk when undergoing surgical embolectomy [6].

Despite the absence of clear guidelines for using thrombolytics in cases of venous thromboembolism in the presence of absolute contraindications, our team decided to administer thrombolytic therapy to our patient. Our case is unique because our patient had a hemorrhagic stroke one day before developing a massive pulmonary embolism complicated by cardiac arrest. The thrombolytic administration did not worsen cerebral bleeding despite having an absolute contraindication per current guidelines.

We searched the literature and found other cases where thrombolytics were administered despite absolute contraindications, and the patient outcomes were positive.

Table 1 shows a list of case reports where thrombolytics were administered in the presence of absolute contraindications, and the patients had positive outcomes [3,7-11]. In some cases, systemic thrombolysis was used successfully in a patient who recently had a hemorrhagic stroke, just like in our case. In other studies, thrombolytics were used in the setting of other absolute contraindications.

**Table 1:** Cases where thrombolytics were administered in the presence of absolute contraindications and patient outcomes. tPA: tissue plasminogen activator; CVA: cerebrovascular accident.

Study reference	Patient biodata		Contraindications for tPA use	Reasons for administration	Thrombolytic agent used	Patient outcome
Bottinor et al. (2014) [3]	60 years, F		Hemorrhagic CVA	Pulmonary embolus	Alteplase	Survived
Koroneos et al. (2007) [7]	53 years, M		Intracerebral hemorrhage	Pulmonary embolus	Alteplase	Survived
Saleh Velez et al. (2021) [8]	75 years, F		Ischemic CVA	Pulmonary embolus	Catheter-directed thrombolysis	Survived
Naidoo et al. (2011) [9]	38 years, F		Ischemic CVA	Pulmonary embolus	Streptokinase	Survived
Pelletier et al. (2010) [10]	35 years, F		Paradoxical stroke	Pulmonary embolus	Alteplase	Survived
Han et al. (2006) [11]		77 years, M	Glioblastoma multiforme	Pulmonary embolus	Alteplase	Survived

In summary, managing massive pulmonary embolism in patients with recent intracranial hemorrhage complicated by cardiac arrest is challenging, and there are no clear guidelines on how to proceed. Despite having absolute contraindications, our decision to administer thrombolytic therapy in our patient's case was successful and did not worsen the cerebral bleeding.

## Conclusions

The mortality associated with massive PE is significantly high and in selected cases, the risk of poor outcome secondary to intracerebral hemorrhage might be outweighed by the high risk of death without thrombolytic therapy.

The cases highlighted in this report elucidate the need for disease-specific guidelines and contraindications, particularly in cases of venous thromboembolism. It further advocates for a case-by-case approach when dealing with venous thromboembolism management, even though absolute contraindications to thrombolytics exist.
